# Neural Hyperactivity Is a Core Pathophysiological Change Induced by Deletion of a High Autism Risk Gene *Ash1L* in the Mouse Brain

**DOI:** 10.3389/fnbeh.2022.873466

**Published:** 2022-04-05

**Authors:** Yuen Gao, Mohammad B. Aljazi, Jin He

**Affiliations:** Department of Biochemistry and Molecular Biology, College of Natural Science, Michigan State University, East Lansing, MI, United States

**Keywords:** autism spectrum disorder, intellectual disability, epilepsy, excitation/inhibition imbalance, neural hyperactivity, *ASH1L*

## Abstract

*ASH1L* is one of the highest risk genes associated with autism spectrum disorder (ASD) and intellectual disability (ID). Our recent studies demonstrate that loss of *Ash1l* in the mouse brain is sufficient to induce ASD/ID-like behavioral and cognitive deficits, suggesting that disruptive *ASH1L* mutations are likely to have a positive correlation with ASD/ID genesis. However, the core pathophysiological changes in the *Ash1l*-deficient brain remain largely unknown. Here we show that loss of *Ash1l* in the mouse brain causes locomotor hyperactivity, high metabolic activity, and hyperactivity-related disturbed sleep and lipid metabolic changes. In addition, the mutant mice display lower thresholds for the convulsant reagent-induced epilepsy and increased neuronal activities in multiple brain regions. Thus, our current study reveals that neural hyperactivity is a core pathophysiological change in the *Ash1l*-deficient mouse brain, which may function as a brain-level mechanism leading to the *Ash1l*-deletion-induced brain functional abnormalities and autistic-like behavioral deficits.

## Introduction

Autism spectrum disorder (ASD) is a neurodevelopmental disorder that has impaired sociability and stereotyped behaviors as its core clinical manifestations, and seizures, intellectual disability, anxiety, attention deficiency, and hyperactivity as its common comorbidities (Hodges et al., 2020). Previous studies have revealed that ASD has a strong genetic basis and is associated with various genetic variants (Lord et al., 2020). Recent whole genome/exome sequencing studies on large patient cohorts identify more than one hundred ASD risk genes that are enriched with gene functions in chromatin modifications and transcriptional regulation (Stessman et al., 2017; Grove et al., 2019; Ruzzo et al., 2019; Lalli et al., 2020; Satterstrom et al., 2020). Notably, *ASH1L* (*A*bsent, *S*mall, or *H*omeotic discs *1*-*L*ike), a gene encoding a histone H3 lysine 36 (H3K36)-specific methyltransferase, is identified as one of the highest ASD risk genes (Stessman et al., 2017; Grove et al., 2019; Ruzzo et al., 2019; Satterstrom et al., 2020). The genetic findings are further supported by multiple clinical cases reporting that some children diagnosed with ASD and/or ID acquire *de novo* disruptive or missense mutations of *ASH1L* (de Ligt et al., 2012; Wang et al., 2016; Okamoto et al., 2017; Faundes et al., 2018; Shen et al., 2019; Xi et al., 2020). In addition to ASD and ID, patients also have a variety of developmental abnormalities (de Ligt et al., 2012; Okamoto et al., 2017; Shen et al., 2019), suggesting its critical roles in normal embryonic and postnatal development.

To determine the relationship between disruptive *ASH1L* mutations in ASD/ID genesis, we generated an *Ash1l* conditional knockout (*Ash1l*-cKO) mouse line and deleted *Ash1l* in the mouse brain by crossing the *Ash1l*-cKO mice with Nestin-Cre mice (*Ash1l*-Nes-cKO). The Cre recombinase expressed in the Nestin-positive neural progenitor cells (NPCs) induced *Ash1l* deletion in NPCs and NPC-derived neuronal and glial lineages, thus leading to *Ash1l* loss in the mouse brain. Using this mouse model, we reported that loss of *Ash1l* in the mouse brain was sufficient to cause multiple developmental defects, core autistic-like behaviors, and ID-like deficits in cognitive memory, suggesting a possible correlation between disruptive *ASH1L* mutations and some core symptoms of ASD/ID (Gao et al., 2021). However, the key pathophysiological changes in the *Ash1l*-deficient brain, which is essential for further characterizing the underlying cellular and molecular mechanisms in the *Ash1l*-mutation-induced ASD pathogenesis, remain largely unknown.

In this study, we used the *Ash1l*-deletion-induced ASD mouse model to investigate various behavioral and physiological changes commonly observed in human ASD patients. Our results show that loss of *Ash1l* in the mouse brain caused locomotor and metabolic hyperactivity, reduced thresholds for the convulsant reagent-induced seizures, and increased neuronal activities in multiple brain regions. The collective results indicate that neural hyperactivity is a core pathophysiological change in the *Ash1l*-deficient brain, which may function as a brain-level mechanism leading to the autistic-like behavioral deficits in the *Ash1l*-deficient mice.

## Materials and Methods

### Mice

The *Ash1l* conditional knockout mice were described in a previous report (Gao et al., 2021). Mice were housed under standard conditions (12 h light: 12 h dark cycles) with food and water *ad libitum*. All mouse experiments were performed with the approval of Michigan State University Institutional Animal Care and Use Committee.

### Mouse Breeding Strategy

Generating *Ash1l*-Nestin-cKO mice: The *Ash1l* neural conditional knockout mice were generated by mating *Ash1l* floxed mice with Nestin-cre mice [B6.Cg-Tg (Nes-cre) 1Kln/J, The Jackson Laboratory]. The wild-type (*Ash1l*^2f/2f^;Nestin-Cre^–/–^), heterozygous (*Ash1l*^2f/+^; Nestin-Cre^±^), and homozygous *Ash1l*-Nes-cKO (*Ash1l*-Nes-cKO, *Ash1l*^2f/2f^;Nestin-Cre^±^) were generated by *Ash1l*^2f/2f^;Nestin-Cre^–/–^ (female) x *Ash1l*^2f/+^;Nestin-Cre^±^ (male) mating.

### Genotyping

Genomic DNA was extracted from mouse tails with a lysis buffer of 0.01 M NaOH. After neutralizing with Tris-HCl (PH 7.6), the extracted genomic DNA was used for genotyping PCR assays. Primers used for genotyping are the same as reported previously (Gao et al., 2021).

### Histology

Mouse adipose and liver tissues were fixed in 4% PFA in PBS at 4°C overnight and embedded in paraffin. For histology analysis, sections of 5 μm were stained with hematoxylin and eosin after dewaxing and rehydration. Images were captured using a Zeiss Axio Imager microscope (Carl Zeiss GmbH, Oberkochen, Germany) and an AxioCam HRc camera (Carl Zeiss GmbH, Oberkochen, Germany). Adipocyte diameter and lipid droplet diameter were measured via Zeiss Zen Pro software (v.2.3; Carl Zeiss GmbH).

### Measurement of Locomotor and Metabolic Activity

Metabolic phenotyping and home cage activity were measured in TSE cages (PhenoMaster, TSE Systems) at Metabolic Core of Michigan State University. Metabolic cage measurement is conducted continuously for 72 h to account for acclimation of mice housed in home cages during this study. Both adult (∼4-month-old) male and female mice were continuously monitored for food and water intake, locomotor activity, and energy expenditure. The locomotor activity was measured by the total times that mice passed through the infrared sensor and total running distances in their home cages. Ambient temperature was maintained at 20–23°C, and the airflow rate through the chambers was adjusted to maintain an oxygen differential around 0.3% at resting conditions. Metabolic parameters including VO_2_, VCO_2_, respiratory exchange ratio, and energy expenditure were assessed via indirect calorimetry by comparing O_2_ and CO_2_ concentrations relative to a reference cage.

### Immuno-Histological Assays

Mouse tissues were fixed in 4% PFA in PBS overnight at 4°C and embedded in paraffin. For immunofluorescence, tissue sections of 5 μm were cut, dewaxed, and rehydrated. Antigen retrieval was performed by microwaving the sections in 0.01 M sodium citrate buffer (pH 6.0) for 4 min. Tissue sections were blocked in 5% normal donkey serum (NDS) for 30 min after washing with PBS. Tissue sections were then incubated with goat anti-Fos (1:300, sc-52-G, Cell Signaling technology) diluted in 5% NDS overnight at 4°C. After washing with PBS, sections were incubated with Rhodamine (TRITC) AffiniPure donkey anti-goat IgG (1:300, Jackson ImmunoResearch Laboratories) for 1 h and mounted using Vectorshield mounting media with DAPI (H1200, Vector Laboratories). Images were captured using a Zeiss Axio Imager microscope (Carl Zeiss GmbH, Oberkochen, Germany) and an AxioCam HRc camera (Carl Zeiss GmbH, Oberkochen, Germany) with image acquisition via Zeiss Zen Pro software (v.2.3; Carl Zeiss GmbH). ∼0.25 mm^2^ frame was used to gate the brain area for counting the c-Fos positive cells.

### Pentylenetetrazole Administration

Pentylenetetrazole (PTZ) (Sigma-Aldrich) was dissolved in PBS at the concentration of 20 mg/ml and administrated through intraperitoneal injection (40 mg/kg).

### Electroencephalography Recording

Two-month-old mice were used for the electroencephalography (EEG) recording, *n* = 3 males and three females for each genotype. Each animal was implanted with three recording electrodes placed at +1.50 A/P, +1.50 L; +1.50 A/P, −1.50 L; −2.0 A/P. +3.00 L, one ground electrode placed at −6.00 A/P, +0.50 L and one reference electrode placed at −3.5 A/P, +3.50 L relative to bregma. 14 days after operation, EEG was recorded on freely moving mice for 30 min before and after the PTZ (40 mg/kg, i.p.) injection. The EEG data was recorded in the Neurologger2A (Evolocus LLC, Tarrytown, NY, United States). The EEG was visualized by EDFbrower version 1.88.

### Statistical Analysis

All statistical analyses were performed using GraphPad Prism 8 (GraphPad Software). Parametric data were analyzed by a two-tailed *t*-test or two-way ANOVA test for comparisons of multiple samples. *P*-values < 0.05 were considered statistically significant. Planned comparisons (Šídák’s multiple comparisons test) were used if ANOVAS showed significant main or interaction effects. Data are presented as mean ± SEM.

## Results

### Loss of *Ash1L* in the Mouse Brain Causes Locomotor Hyperactivity

**FIGURE 1:**
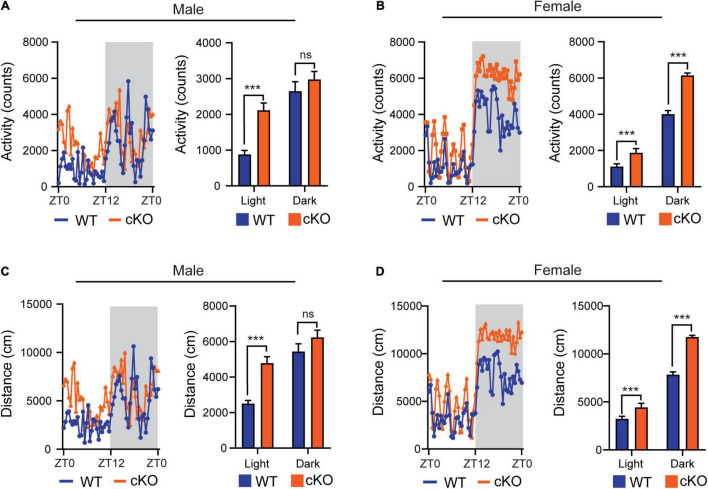
Loss of *Ash1L* in the mouse brain causes locomotor hyperactivity. **(A,B)** Plots showing total times (counts) that male **(A)** and female **(B)** wild-type and *Ash1l*-Nes-cKO mice passing through an infrared sensor in a 12-h light-on cycle (ZT0–ZT12, Zeitgeber Time) and a 12-h light-off cycle (ZT12–ZT0, Zeitgeber Time). *n* = 4 males and four females per genotype. *P*-values calculated using two-way ANOVA tests. Error bars in graphs represent mean ± SEM. ***p < 0.001; ns, not significant. **(C,D)** Plots showing the distances that male **(C)** and female **(D)** wild-type and *Ash1l*-Nes-cKO mice run in the 12-h light-on cycle (ZT0 – ZT12, Zeitgeber Time) and the 12-h light-off cycle. *n* = 4 males and four females per genotype. *P*-values calculated using two-way ANOVA tests. Error bars in graphs represent mean ± SEM. ***p < 0.001; ns, not significant.

In the previous open field tests, we observed that the mutant mice (*Ash1l*-Nes-cKO) had high locomotor activity compared to their wild-type littermates (Gao et al., 2021). Since the mutant mice also displayed increased anxiety in the open field test chambers (Gao et al., 2021), which may induce locomotor hyperactivity, we used the TSE PhenoMaster/LabMaster System to measure the locomotor activity and total running distances in their home cages (Van Klinken et al., 2012). The results showed that except for the male in the light-off cycle (ZT12-ZT0, Zeitgeber Time), both male and female mutant mice had significantly more locomotor activity [male, *F*_(1,52)_ = 29.56, light cycle *p*<0.001, dark cycle *p* = 0.319; female, *F*_(1,52)_ = 259.0, light cycle *p*<0.001, dark cycle *p*<0.001] and longer running distances [male, *F*_(1,52)_ = 27.45, light cycle *p*<0.001, dark cycle *p* = 0.1112; female, *F*_(1,52)_ = 257.6, light cycle *p*<0.001, dark cycle *p*<0.001] (Figure 1), indicating their active movement in home cages. Notably, compared to wild-type controls, both male and female mutant mice maintained higher locomotor activities during the light-on cycle (ZT0-ZT12) in which wild-type mice spent most time in sleep and maintained relatively low locomotor activity, suggesting that the sleep of mutant mice was greatly disturbed by their high locomotor activity.

### Loss of *Ash1l* in the Mouse Brain Causes Metabolic Hyperactivity

**FIGURE 2:**
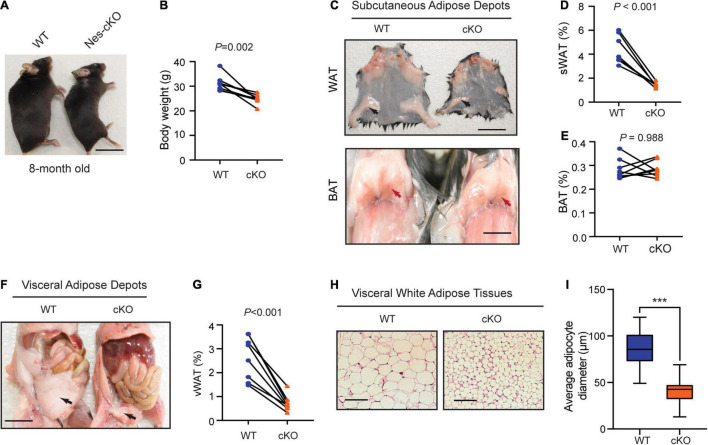
Loss of *Ash1l* in the mouse brain reduces adipose tissue depots. **(A)** Representative photos showing the low body weight of adult *Ash1l*-Nes-cKO mice compared to wild-type littermates. **(B)** Plot showing the body weight of adult (8-month-old) wild-type and *Ash1l*-Nes-cKO mice. For each group, *n* = 8. *P*-values calculated using paired *t*-tests. **(C)** Photos showing subcutaneous white adipose depots (WAT) and brown adipose deports (BAT) in wild-type and *Ash1l*-Nes-cKO adult mice. Bar = 0.5 cm. **(D,E)** Plots showing the quantitative measurement of subcutaneous white adipose depots (sWAT) and brown adipose depots (BAT) in wild-type and *Ash1l*-Nes-cKO mice. Y-axis represents the percentage of subcutaneous adipose tissue normalized to total body weight. For each group, *n* = 8. *P*-values calculated using paired *t*-tests. **(F)** Representative photos and showing the visceral white adipose depots in wild-type and *Ash1l*-Nes-cKO mice. Bar = 0.5 cm. **(G)** Plots showing the quantitative measurement of visceral white adipose depots (vWAT) in wild-type and *Ash1l*-Nes-cKO mice. Y-axis represents the percentage of visceral adipose tissue normalized to total body weight. For each group, *n* = 8. *P*-values calculated using paired *t*-tests. **(H)** Photos showing the adipocytes of visceral white adipose tissues under microscope. Bar = 50 μm. **(I)** Plot showing the average diameters of adipocytes of wild-type and *Ash1l*-Nes-cKO mice. For each group, *n* = 30. *P*-values calculated using unpaired *t*-tests. ***p < 0.001.

Compared to wild-type littermates, adult mutant mice had significantly lower body weight (*t* = 4.688, df = 7, *p* = 0.0022) (Figures 2A,B), and markedly reduced subcutaneous (*t* = 7.259, df = 7, *p* = 0.002) and visceral adipose tissue depots (*t* = 7.180, df = 7, *p* = 0.002), which appeared to mainly affect white adipose tissues (WATs), a major form of adipose tissues for triacylglycerol storage (Saely et al., 2012), but not brown adipose tissues (BATs) (*t* = 0.0150, df = 7, *p* = 0.9884) (Figures 2C–G). Histological analyses showed that the WATs in the mutant mice had smaller cell sizes (*t* = 11.49, df = 58, *p* < 0.0001) but maintained similar cell numbers (Figures 2H,I), suggesting that reduced WATs in the mutant mice were likely to be caused by energy over-expenditure and reduced triacylglycerol storage in WATs but not due to the loss of adipocytes.

**FIGURE 3:**
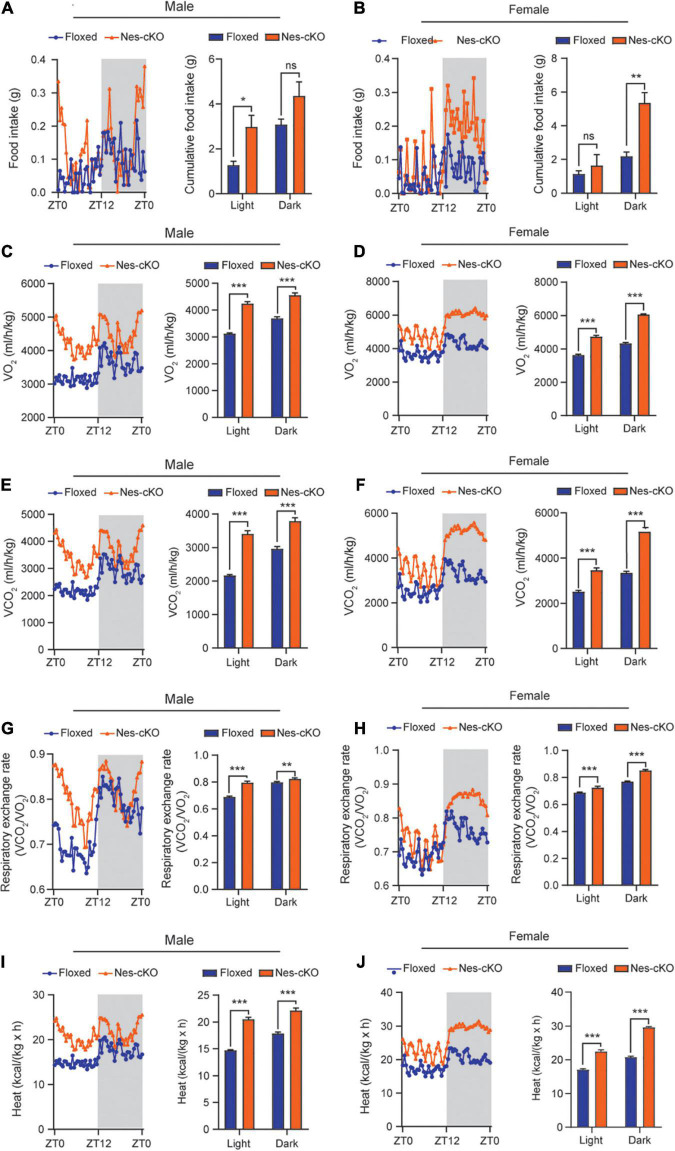
Loss of *Ash1l* in the mouse brain causes metabolic hyperactivity. **(A,B)** Plots showing the food intake of male **(A)** and female **(B)** wild-type and *Ash1l*-Nes-cKO mice in the light-on cycle (ZT0–ZT12, Zeitgeber Time) and in light-off cycle (ZT12–ZT0). *n* = 4 males and four females per genotype. *P*-values calculated using two-way ANOVA tests. Error bars in graphs represent mean ± SEM. *p < 0.05; **p < 0.01; ns, not significant. **(C–J)** Plots showing the oxygen (VO_2_) consumption **(C,D)**, carbon dioxide (VCO_2_) production **(E,F)**, respiration exchange ratio (VCO_2_/VO_2_) **(G,H)**, and heat generation **(I,J)** of wild-type and *Ash1l*-Nes-cKO mice in the 12-h light-on cycle (ZT0–ZT12, Zeitgeber Time), and in the 12-h light-off cycle (ZT12–ZT0, Zeitgeber Time). *n* = 4 males and four females per genotype. *P*-values calculated using two-way ANOVA tests. Error bars in graphs represent mean ± SEM. **p < 0.01; ***p < 0.001.

Generally, reduced adipose tissue depots could be resulted from either insufficient food intake or increased energy expenditure (Kahn et al., 2019). To differentiate these two underlying causes, we used the TSE PhenoMaster/LabMaster System to measure food intake and energy expenditure including oxygen consumption (VO_2_), carbon dioxide production (VCO_2_), respiration exchange ratio (VCO_2_/VO_2_), and heat generation. The results showed that both male and female mutant mice had higher or similar food intake compared to their wild-type littermates [male, *F*_(1,330)_ = 25.48, light cycle *p* = 0.0322, dark cycle *p* = 0.1150; female, *F*_(1,330)_ = 39.45, light cycle *p* = 0.7243, dark cycle *p* = 0.001] (Figures 3A,B). Consistent with the locomotor hyperactivity, the mutant mice had higher oxygen consumption [male: *F*_(1,330)_ = 419.0, light cycle *p*<0.001, dark cycle *p*<0.001; female: *F*_(1,330)_ = 762.4, light cycle *p*<0.001, dark cycle *p*<0.001], carbon dioxide production [male: *F*_(1,330)_ = 361.4, light cycle *p*<0.001, dark cycle *p*<0.001; female: *F*_(1,330)_ = 668.9, light cycle *p*<0.001, dark cycle *p*<0.001], respiration exchange ratios [male: *F*_(1,330)_ = 140.6, light cycle *p*<0.001, dark cycle *p*<0.01; female: *F*_(1,330)_ = 149.9, light cycle *p*<0.001, dark cycle *p*<0.001], and heat generation [male: *F*_(1,330)_ = 409.1, light cycle *p*<0.001, dark cycle *p*<0.001; female: *F*_(1,330)_ = 754.2, light cycle *p*<0.001, dark cycle *p*<0.001] (Figures 3C–J), suggesting loss of *Ash1l* in the brain caused overall metabolic hyperactivity and high calorie consumption. Same as the locomotor hyperactivity, the higher metabolic activity of mutant mice appeared to be more prominent during the light-on phase (ZT0-ZT12) in which wild-type mice spent most time in sleep and had a relatively low metabolic rate (Figure 3), further suggesting that the normal circadian cycle and sleep were disturbed in the mutant adult mice due to their high locomotor and metabolic activities.

### Loss of *Ash1l* in the Mouse Brain Reduces the Thresholds for the Pentylenetetrazole-Induced Epilepsy

**FIGURE 4:**
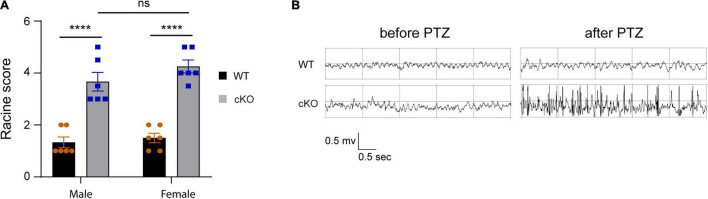
Loss of *Ash1l* in the mouse brain reduces the thresholds for the PTZ-induced epilepsy. **(A)** Plots showing the Racine score of epileptic behaviors of male and female wild-type and *Ash1l*-Nes-cKO mice. *n* = 6 males and six females per genotype. *P-*value calculated using two-way ANOVA tests. Error bars in graphs represent mean ± SEM. ****p < 0.0001. **(B)** Representative electroencephalography (EEG) recording showing the electrical waves before and after sub-convulsant dose of pentylenetetrazole (PTZ) injection.

Previous studies have shown that ASD patients with *ASH1L* mutations have a high occurrence of epilepsy (Wang et al., 2016; Shen et al., 2019). To examine whether the mice with *Ash1l* loss in the brain were more likely to develop epilepsy, we challenged both wild-type and mutant mice by intraperitoneal injection of PTZ, a GABA_A_ receptor antagonist, at a sub-convulsant dose (40 mg/kg) to induce seizures (Sansig et al., 2001). The thresholds for the PTZ-induced seizures were measured by scoring epileptic behaviors in 10 min after PTZ administration according to a previous report (Van Erum et al., 2019). The results showed that the sub-convulsant dose of PTZ induced minor epileptic behaviors such as moving arresting, whisker trembling, and facial jerking in wild-type mice. In contrast, both male and female mutant mice displayed much more severe epileptic behaviors including heavy myoclonic jerks, lying on belly with rapid body twitches, and clonic-tonic spasm [genotype effect: *F*_(1,10)_ = 453.8, *p*<0.001; sex effect: *F*_(1:10)_ = 1.17, *p* = 0.3], suggesting that the mutant mice had lower thresholds for the PTZ-induced epilepsy (Figure 4A and Supplementary Videos 1, 2). Moreover, EEG recordings on frontal cortices showed that although both wild-type and mutant mice had reduced electric wave frequency after PTZ administration, the mutant mice had spike-wave electrical discharges with increased amplitude (Figure 4B), which was consistent with the severe epileptic behaviors observed in the mutant mice. Altogether, the results suggested that loss of *Ash1l* in the mouse brain increased excitability of cortical neurons, which reduced the thresholds for the convulsant reagent-induced seizures.

### Loss of *Ash1l* in the Mouse Brain Induces Neuronal Hyperactivity in Multiple Brain Regions

**FIGURE 5:**
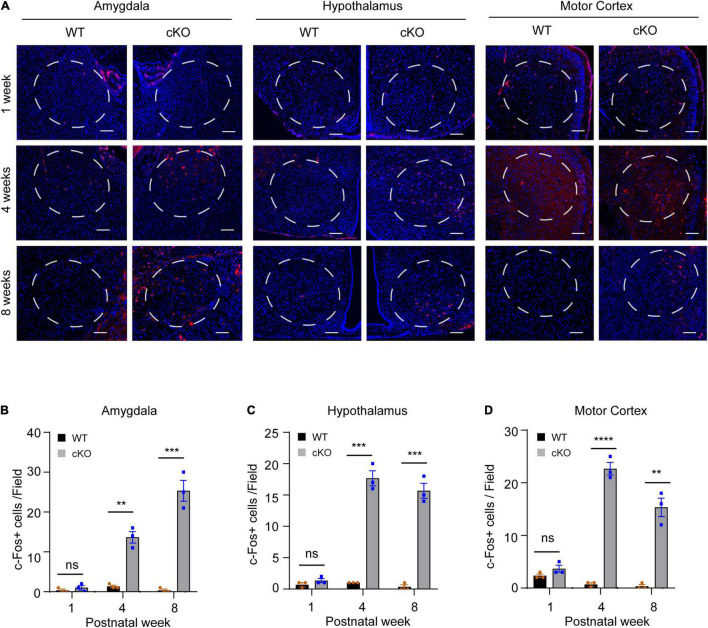
Loss of *Ash1l* in the mouse brain causes neuronal hyperactivity in broad brain regions. **(A)** Representative photos showing the c-Fos positive cells in amygdalae hypothalami, and motor cortices. bar = 100 μm. **(B–D)** Bar plots showing the c-Fos positive cells in amygdalae **(B)**, hypothalami **(C)**, and motor cortices **(D)**. *n* = 3 brain samples/per genotype. The numbers of c-Fos positive cells per brain were calculated as the mean c-Fos positive cell numbers from three sections (∼100 μm apart) examined. A frame (∼0.25 mm^2^) was used to gate the courting area for all sections. *P-*value calculated using two-tailed *t-*tests. Error bars in graphs represent mean ± SEM. **p < 0.01, ***p < 0.001, ****p < 0.0001.

To identify the brain areas involved in locomotor and metabolic hyperactivity in mutant mice, we used the c-Fos immunoreactivity as a neuronal activation marker to screen brain regions with neuronal hyperactivity (Bullitt, 1990). The results showed that both wild-type and mutant pups (postnatal day 7, PN7) had few c-Fos + cells in the brain. However, compared to wild-type mice, young (PN30) and adult (PN60) mutant mice had significant increased c-Fos positive cells in motor cortices (week 4: *t* = 17.64, df = 4, *p* < 00001; week 8: *t* = 8.356, df = 4, *p* = 0.0011), amygdalae (week 4: *t* = 8.273, df = 4, *p* = 0.0012; week 8: *t* = 9.5, df = 4, *p* = 0.0007), and hypothalami (week 4: *t* = 13.87, df = 4, *p* = 0.0002; week 8: *t* = 12.29, df = 4, *p* = 0.0003) (Figures 5A–D), suggesting that high neuronal activity gradually developed in multiple cortical and subcortical regions in the postnatal *Ash1l*-deficient brain.

## Discussion

Recent genetic and clinical studies report that *ASH1L* is an ASD high risk gene. To examine the relationship between disruptive *ASH1L* mutations and ASD genesis, we recently used an *Ash1l* conditional knockout mouse model to show that loss of *Ash1l* in the mouse brain was sufficient to induce ASD/ID-like behavioral and cognitive deficits, suggesting disruptive *ASH1L* mutations are likely to have a positive correlation with some core symptoms of ASD (Gao et al., 2021). However, the key pathophysiological changes leading to the brain functional abnormities and autistic-like behavioral deficits in the *Ash1l*-deficient mice are largely unclear.

In this study, we provide multiple lines of evidence to show that neural hyperactivity is a core pathophysiological change in the *Ash1l-*deficient mouse brain. First, consistent with the observation that the *Ash1l* mutant mice had longer running distances in the open field test (Gao et al., 2021), the 72-h locomotor activity measurement showed that compared to wild-type controls, the *Ash1l* mutant mice had more active movement and longer running distances in their home cages (Figure 1), suggesting that the locomotor hyperactivity of *Ash1l* mutant mice was likely to be an outcome of *Ash1l* loss in the brain but not due to increased anxiety in the open field test chamber. Second, adult *Ash1l* mutant mice had lower body weight and markedly reduced white adipose tissue depots (Figure 2). The systemic measurement of metabolic activity demonstrated that both male and female *Ash1l* mutant mice had increased food intake and metabolic hyperactivity (Figure 3), suggesting the lower body weight and the reduced adipose tissue depots in the *Ash1l* mutant mice were likely to be caused by metabolic hyperactivity-induced lipid catabolism but not hypothalamus dysfunction or its-related insufficient food intake. Furthermore, the increased metabolic activity in the mutant mice appeared to be proportional to the high locomotor activity at both light-on and light-off phases (Figures 1, 3), suggesting that the metabolic hyperactivity was likely to be caused by the energy over-expenditure due to high locomotor activity. Notably, the mutant mice maintained both locomotor and metabolic hyperactivity during the normal sleeping phase (Figures 1, 3), indicating that the *Ash1l* mutant mice have difficulties in sleep due to their high activities, which was consistent with the sleeping difficulty commonly observed in ASD patients (Devnani and Hegde, 2015; Elrod and Hood, 2015; Veatch et al., 2015). Third, consistent with the observation that ASD patients have a high occurrence of epilepsy (Besag, 2018; Pacheva et al., 2019), we observed that the mutant mice had more severe epileptic behaviors and spike-wave electrical discharges in cortices in response to a sub-convulsant dose of PTZ (Figure 4), suggesting that loss of *Ash1l* in the brain reduces the threshold for seizures triggered by sub-convulsant electrical signals in cortices, which may be caused by reduced inhibitory signals and loss of excitation-inhibition balance in neural circuits. Consistently, compared to wild-type controls, the *Ash1l* mutant mice had gradually increased c-Fos positive neuronal populations in various regions in the postnatal developed brain (Figure 5), suggesting the *Ash1l-*loss-induced neuronal hyperactivity appear to occur in multiple postnatal cortical and subcortical areas but not restricted to specific brain regions.

Although our current study supports a positive correlation between disruptive *Ash1l* mutations and autistic-like behavioral deficits in our mouse model, it is worth to note that our conclusion is largely based on the results from specific behavioral tests such as three-chamber tests in adult animals. Moreover, some hyperactivity-related physiological changes, such as disturbed sleep in mutant mice, were not quantitatively measured. To fully assess the relationship between disruptive *Ash1l* mutations and autistic-like phenotypes in our mouse model, it is necessary to perform additional behavioral tests such as ultrasonic vocalizations, auditory startle responses, and direct social interactions in both young and adult animals (Fyke et al., 2021a,b, c). In addition, measurements of sleeping cycles by polysomnography are needed to quantify the disturbed sleep in mutant mice (Tagaito et al., 2001).

An increased ratio of excitatory versus inhibitory (E/I) neural signals has been commonly found in ASD associated with a variety of genetic variants (Uzunova et al., 2016; Goel and Portera-Cailliau, 2019). It has been postulated that loss of E/I balance interferes the development and maturation of neural networks in the developing brain, which impairs normal brain functions and leads to ASD behavioral deficits (Rubenstein and Merzenich, 2003; Sohal and Rubenstein, 2019). Consistent with this hypothesis, our current study demonstrates that loss of *Ash1l*, one of the highest ASD risk genes identified in human patients, leads to neural hyperactivity and its-related behavioral and physiological changes in mice. Although the mechanisms underlying the *Ash1l*-mutation-induced ASD genesis remain largely unelucidated, the identification of neural hyperactivity as a core pathophysiological change in the *Ash1l*-deficient mice provides a brain-level basis for further dissecting the molecular and cellular components contributing to the E/I imbalance and autistic behavioral deficits in the *ASH1L-*mutation-related ASD.

## Data Availability Statement

The original contributions presented in the study are included in the article/Supplementary Material, further inquiries can be directed to the corresponding author.

## Ethics Statement

The animal study was reviewed and approved by Michigan State University Institutional Animal Care and Use Committee.

## Author Contributions

JH conceived and oversaw the project. YG performed the experiments. YG and MA maintained the mouse colonies. YG and JH interpreted the data and wrote the manuscript. All authors contributed to the article and approved the submitted version.

## Conflict of Interest

The authors declare that the research was conducted in the absence of any commercial or financial relationships that could be construed as a potential conflict of interest.

## Publisher’s Note

All claims expressed in this article are solely those of the authors and do not necessarily represent those of their affiliated organizations, or those of the publisher, the editors and the reviewers. Any product that may be evaluated in this article, or claim that may be made by its manufacturer, is not guaranteed or endorsed by the publisher.
